# Adherence to a traditional Mexican diet and non-communicable disease-related outcomes: secondary data analysis of the cross-sectional Mexican National Health and Nutrition Survey

**DOI:** 10.1017/S0007114522002331

**Published:** 2023-04-14

**Authors:** Selene Valerino-Perea, Miranda E. G. Armstrong, Angeliki Papadaki

**Affiliations:** Centre for Exercise, Nutrition and Health Sciences, School for Policy Studies, University of Bristol, Bristol, BS8 1TZ, UK

**Keywords:** Traditional diets, Non-communicable diseases, Obesity, Dietary patterns, Traditional Mexican diet

## Abstract

This study evaluated the association between adherence to a traditional Mexican diet (TMexD) and obesity, diabetes and CVD-related outcomes in secondary data analysis of the cross-sectional Mexican National Health and Nutrition Survey 2018–2019. Data from 10 180 Mexican adults were included, collected via visits to randomly selected households by trained personnel. Adherence to the TMexD (characterised by mostly plant-based foods like maize, legumes and vegetables) was measured through an adapted version of a recently developed TMexD index, using FFQ data. Outcomes included obesity (anthropometric measurements), diabetes (biomarkers and diagnosis) and CVD (lipid biomarkers, blood pressure, hypertension diagnosis and CVD event diagnosis) variables. Percentage differences and OR for presenting non-communicable disease (NCD)-related outcomes (with 95 % CI) were measured using multiple linear and logistic regression, respectively, adjusted for relevant covariates. Sensitivity analyses were conducted according to sex, excluding people with an NCD diagnosis and using multiple imputation. In fully adjusted models, high, compared with low, TMexD adherence was associated with lower insulin (−9·8 %; 95 % CI (−16·0, −3·3)), LDL-cholesterol (−4·3 %; 95 % CI (−6·9, −1·5)), non-HDL-cholesterol (−3·9 %; 95 % CI (−6·1, −1·7)) and total cholesterol (−3·5 %; 95 % CI (−5·2, −1·8)) concentrations. Men and those with no NCD diagnosis had overall stronger associations. Effect sizes were smaller, and associations weakened in multiple imputation models. No other associations were observed. While results may have been limited due to the adaptation of a previously developed index, the results highlight the potential association between the TMexD and lower insulin and cholesterol concentrations in Mexican adults.

Traditional diets refer to long-established food patterns that represent a region’s food culture^(1)^. Given that these diets generally contain large amounts of plant-based and non-industrialised foods^(1,2)^, consuming certain traditional diets (e.g. the Mediterranean diet) has been recommended for preventing non-communicable diseases (NCD)^(3–6)^, also known as chronic diseases, such as diabetes and CVD^(7)^. In addition, traditional diets have been recognised as environmentally friendly and culturally appropriate nutrition strategies, which are public health priorities set by global health institutions^(3,4,8)^. However, not all traditional diets follow all nutrient recommendations in current food guidelines and therefore must be evaluated in relation to health before their promotion. For instance, the traditional Mexican diet (TMexD) may contribute to better health outcomes through high intakes of plant-based foods (e.g. maize, legumes, vegetables, grains, fruits and seeds)^(9–11)^. These foods, rich in dietary fibre, diverse micronutrients and antioxidants^(12)^, have been associated with reduced body weight^(13–15)^, glucose^(16)^, insulin^(16)^, blood pressure^(17)^ and some types of blood cholesterol levels^(17,18)^. However, the TMexD is also abundant in items incompatible with current food guidelines, like energetic beverages and energy-dense dishes (e.g. *tamales*)^(11)^.

In Mexico, the TMexD must be evaluated in relation to obesity, diabetes and CVD, which are outcomes of major public health interest, before it is promoted in any nutrition strategies. Studying the link between traditional diets and the high burden of disease in Mexico is particularly important to study, as prevalence rates of obesity and diabetes are amongst the highest worldwide^(19)^ (36·1 % and 13·7 %, respectively)^(20,21)^, while CVD remain the leading cause of death in the country (22·7 %)^(22)^. This high burden of disease has been attributed to the population’s poor adherence to dietary guidelines, with few Mexican adults meeting recommended intakes of protective foods (e.g. fruits and vegetables) and most exceeding the recommendations for foods high in energy, fat and added sugars (e.g. sugar-sweetened beverages (SSB))^(23)^.

To date, no studies have explored the association between adherence to a TMexD diet and NCD outcomes in Mexico^(11)^. Previous studies have evaluated the associations between health outcomes and the TMexD using *a posteriori* analyses of the diet^(24–28)^, which provide an evaluation of the population’s current dietary intakes^(29)^ but do not necessarily reflect a traditional diet. Similarly, other studies have used Mexican diet indices by measuring the consumption of a range of foods^(30,31)^; however, these indices have generally omitted potentially relevant foods (i.e. beverages, herbs and condiments, or nuts and seeds), which are typical of the Mexican food culture^(11)^. Using a comprehensive, *a priori* and evidence-based TMexD index to evaluate its association with health outcomes could therefore provide essential evidence on the importance of this traditional dietary pattern, before implementing public health efforts to promote it to the wider Mexican population.

A comprehensive index to measure adherence to the TMexD was recently created, using systematic reviewing^(11)^ and subsequently Delphi methodologies^(10)^. The latter employed expert opinion to select the foods and food-related habits that reflect the TMexD^(32,33)^. The current study aimed to utilise this recently developed TMexD index^(10)^ to investigate the association between TMexD adherence and anthropometric characteristics, and with diabetes and CVD biomarkers and prevalence, in a representative sample of Mexican adults. It was hypothesised that higher TMexD adherence would be associated with more favourable outcomes for obesity, diabetes and CVD, compared with low adherence.

## Materials and methods

### Study design

This study consisted of secondary data analyses of the National Health and Nutrition Survey (ENSANUT) 2018–2019 in Mexico. ENSANUT is a cross-sectional survey with a probabilistic, multi-stage, stratified cluster sampling design, representative at a national level^(34,35)^. Data are publicly available on ENSANUT’s website^(36)^. The data in the present study are reported using the ‘Strengthening the Reporting of Observational studies in Epidemiology’ (STROBE)^(37)^ and the ‘STROBE-Nutritional Epidemiology’ (STROBE-nut)^(38)^ statements (online Supplementary materials I, Table S1).

### Ethical approval

This study was conducted according to the guidelines laid down in the Declaration of Helsinki, and all procedures involving human subjects were approved by the Mexican National Institute for Public Health Institutional Review Board. The School for Policy Studies Research Ethics Committee (SPSREC/18-19/053) at the University of Bristol approved the current study. Written informed consent was obtained from all subjects^(35)^.

### Data sources

Data from ENSANUT 2018–2019 were collected from June 2018 to July 2019 through visits to 50 654 randomly selected households (with an 87 % response rate). Specific information about the sample size calculation is reported elsewhere^(34)^. At least one adult in each household was interviewed; no dietary or physiological characteristics were considered when selecting participants. Data were obtained face to face by trained personnel using standardised procedures^(35)^. Sociodemographic and health data were obtained from 84 490 individuals; anthropometric measurements, blood biomarkers, physical activity and dietary data were obtained from random subsamples^(34,39)^.

The current study included analyses in adults aged 20 to 69 years who completed all questionnaires (older adults (>69 years) were excluded as different measures for some outcomes are reported for this population). Pregnant and lactating women (including women <50 years with missing data for this variable), individuals with implausible health outcomes, and participants with low or extreme dietary intakes were excluded as follows. Participants with implausible health outcomes were those with unlikely values for height (<1·30 m or >2·0 m), BMI (<10 kg/m^2^ or >58 kg/m^2^), waist circumference (<50 cm or >200 cm), systolic blood pressure (<80 mmHg) and diastolic blood pressure (<50 mmHg)^(40)^. Individuals with low dietary intakes were those with an energy intake/BMR ratio below 0·5, according to the Mifflin-St Jeor equation^(41)^ (individuals with missing anthropometric data were assigned the mean BMR by sex group^(42)^). Those with high total energy intakes (TEI) were defined as those with TEI greater than 3 sd from the mean by sex group. Given the use of blood biomarker analyses, individuals with less than 8 h of fasting at the time of the interview were also excluded^(21,43,44)^.

### Dietary variables

Dietary data were collected using a validated^(45)^, interviewer-administered, semi-quantitative FFQ. This FFQ included the 140 most consumed foods in Mexico and those of particular public health interest (relevant to NCD development, such as processed foods, dressings, SSB and full-fat dairy products)^(40,42)^. Participants reported the times per week, times per d, number of portions and portion size of each food consumed during the 7 d before the interview^(40)^. The portion sizes were calculated using the FFQ standard and alternative portions sizes, expressed using home measurements (e.g. pieces of fruit, spoons and cups)^(42)^.

Daily grams and TEI were calculated from weekly intakes by calculating the grams per portion and energy densities reported in the ENSANUT 2012 database. This database, alongside this particular FFQ, was generated by the Mexican National Institute for Public Health^(42)^. For the purposes of the current study, implausible daily intakes (i.e. those greater than 4 sd from the mean by sex, area and region groups) were calculated for each food item in the questionnaire. Participants with seven or more implausible intakes were excluded from the analysis^(40)^. Additional cleaning procedures were applied for measuring tortilla intake, given the large missing values on this variable (as tortilla consumption is reported by reporting the weight of tortillas consumed). For individuals with missing tortilla weight data, each state’s mean weight was calculated and imputed^(46)^ (6·8 % values imputed). Additionally, only intervals of 10–500 g were considered valid tortilla weights. Tortillas weighing <10 g or >500 g were considered implausible and imputed with minimal (10 g) or maximum (500 g) values^(46)^ (0·5 % values imputed).

Adherence to the TMexD was assessed using an adapted version of the TMexD index, recently developed using systematic reviewing and Delphi methodologies^(10,11)^. Briefly, this index was created in a three-round Delphi study, where experts in the TMexD reached a consensus on the items representing a diet traditional of Mexico. The resulting index mainly reflects, according to the participating experts, foods highly consumed in Mexico and past dietary habits^(10)^. This index (score ranging from 0 to 21 points) measures the consumption of fifteen food groups (maize products, legumes, vegetables, fruits, beverages, herbs and condiments, nuts and seeds, vegetable fats and oils, grains, plain water, tubers, meats, dairy products, eggs, and maize-based dishes) and three food-related habits (consuming home-made meals, socialising at meals and buying foods locally) that represent a TMexD (online Supplementary materials II, Table S1). This index was adapted to assess TMexD adherence in the present study; the three components reflecting food-related habits were omitted, as these are not measured in ENSANUT. Foods omitted included items like amaranth, *tostadas*, cacao drinks and vegetable oil, as these are not measured in ENSANUT (online Supplementary materials II, Table S1). The complete list of foods omitted can be found in Supplementary materials II (Table S2). The TMexD index used in the present study consisted of fifteen food groups; scores ranged from 0 to 18, with higher scores representing higher adherence to this traditional diet.

### Outcome variables

Obesity outcomes were assessed using anthropometric measurement variables of weight (kg), height (m) and waist circumference (cm). Data were measured according to international standardised protocols and using calibrated stadiometers and electronic scales^(40)^. All measures were recorded twice; the average was calculated and used for analyses. BMI was calculated using a standard equation (kg/m^2^) and classified as underweight (≤18·4 kg/m^2^), normal weight (18·5–24·9 kg/m^2^), overweight (≥25 kg/m^2^) and obesity (≥30 kg/m^2^)^(47)^.

Diabetes outcomes were measured using glucose, glycated Hb (HbA1c) and insulin concentration values. Diabetes was defined as having either a high fasting plasma glucose (≥126 mg/dl)^(48)^, high HbA1c levels (≥6·5 %)^(48)^ or a previous medical diagnosis of diabetes^(21)^ (not including women diagnosed during pregnancy).

CVD biomarkers measured were blood lipids and blood pressure. The blood lipids used to evaluate CVD risk included LDL-cholesterol, HDL-cholesterol, non-HDL-cholesterol, total cholesterol and TAG. Systolic and diastolic blood pressure measures were recorded twice, and the average was calculated and used for analyses. Hypertension was established in participants with high systolic (>130 mmHg) or diastolic (>80 mmHg) pressure values (according to the updated hypertension guidelines)^(49)^, as well as those with a previous medical diagnosis of hypertension^(50)^ (not including women diagnosed during pregnancy). The occurrence of previously diagnosed CVD events was also assessed via self-report of a previous medical diagnosis of different CVD (i.e. “Has a doctor ever diagnosed you with heart attack, angina or heart failure?”).

### Sociodemographic and other health data

Sociodemographic and health-related data were self-reported and collected using interviewer-administered questionnaires. Sociodemographic data included sex, age, education, area of residence, region of the country and socio-economic status (SES; using the quartiles developed by ENSANUT, which consider household head education, income, access to services and household assets^(51)^). Health data included medication use, family history of disease, smoking status and physical activity (reported in metabolic equivalent task (MET) minutes and assessed using the short form of the International Physical Activity Questionnaire^(52)^).

### Data analysis

#### Statistical analyses

Analyses were performed in Stata version 16.0^(53)^ and using the survey prefix command (SVY) to adjust for the complex survey design^(54)^. The sample characteristics and intakes of food groups in the TMexD were reported using means and proportions. Reporting medians, which is more appropriate for data not normally distributed (e.g. anthropometric and biomarker data)^(55)^, was not possible using the SVY module. The sample characteristics and food intakes were calculated across the categories of the TMexD index using simple linear regression (ANOVA analyses are not available for SVY data) for continuous variables and Pearson’s *χ* ^2^ tests for categorical ones.

The associations between adherence to the TMexD (low, medium and high; classified using tertiles) and continuous outcomes for obesity, diabetes, and CVD risk markers were evaluated using multiple linear regression in complete-case analyses. The assumptions for homoskedasticity, normality and model specification were tested visually and using statistical tests (link test and omitted variable test for model misspecification)^(56)^; all assumptions tested were met. Other regression assumptions (i.e. multicollinearity, constant variance and influential points) were not tested as these are unavailable in the SVY module^(57)^. In all models, the outcome variable was log-transformed to meet these assumptions. Log-transformed results were translated into percentage differences between the highest *v*. the lowest level of TMexD adherence to facilitate interpretation. The association between TMexD adherence (low, medium and high) and the odds for having NCD-related outcomes (yes/no) was tested using multiple logistic regression (in complete-case analyses). The assumption of model specification, the only one available for complex survey-designed data, was tested using statistical tests (link test for model misspecification)^(58)^; all assumptions tested were met. The *P*-value was adjusted (*P* < 0·004) for multiple comparisons using the Bonferroni correction^(59)^.

All analyses were adjusted for confounders identified from a broad literature search. These confounders included age (years; continuous or in categories (20–29, 30–39, 40–49, 50–59, 60–69) based on the model that met regression assumptions), sex (female/male), education (primary or less, secondary, high school, or higher education), SES (quartiles), region (North, Centre-Mexico City and South)^(60–62)^, area of residence (urban/rural), physical activity (MET minutes and continuous) and smoking status (current smoker, previous smoker and never smoker). While there was no difference in TMexD scores by sex, the analyses were adjusted for this variable^(63)^ as earlier studies have found differences in both dietary outcomes and the outcomes evaluated according to sex^(20,64–69)^. For diabetes- and CVD-related outcomes, family history of disease (yes/no) and medication use (yes/no) were also included as confounders; these were added for each condition specifically (e.g. family history of diabetes, diabetes medication use were only used in diabetes-related analyses). While food security has also been associated with dietary intake^(70)^ and health outcomes (e.g. obesity^(20)^, diabetes^(71)^ and hypertension^(71)^) in Mexico, it is not commonly adjusted for and was available in few participants only (55·6 % of the sample), so it was discarded. Since TEI (continuous) or the presence of overweight and obesity (<25 kg/m^2^/ ≥25 kg/m^2^) might affect the association between the outcomes evaluated and the TMexD, the analyses also adjusted for these variables in separate models.

#### Sensitivity analyses

Further sensitivity analyses were conducted. Since studies in Mexican adults have reported sex and education, sex and age, and sex and SES interactions^(72–74)^, all analyses were additionally performed separately by sex. To reduce potential reverse causation bias, individuals previously diagnosed with a chronic disease (i.e. diabetes, hypertension and CVD) or individuals who reported changing their diet after a chronic disease diagnosis (i.e. following a specific diet after diabetes or hyperlipidaemia diagnosis) were excluded in separate sensitivity analyses. Multiple imputation was also conducted to include individuals with incomplete data. Data were imputed using chained equations and twenty imputed datasets and using sociodemographic (i.e. age, sex, education, SES, geographical region and area of residence) and health data (i.e. previous diagnosis of an NCD) as auxiliary variables. The imputed data ranged from 0·9 % to 43·9 %.

## Results

### Participant characteristics

**Fig. 1.**
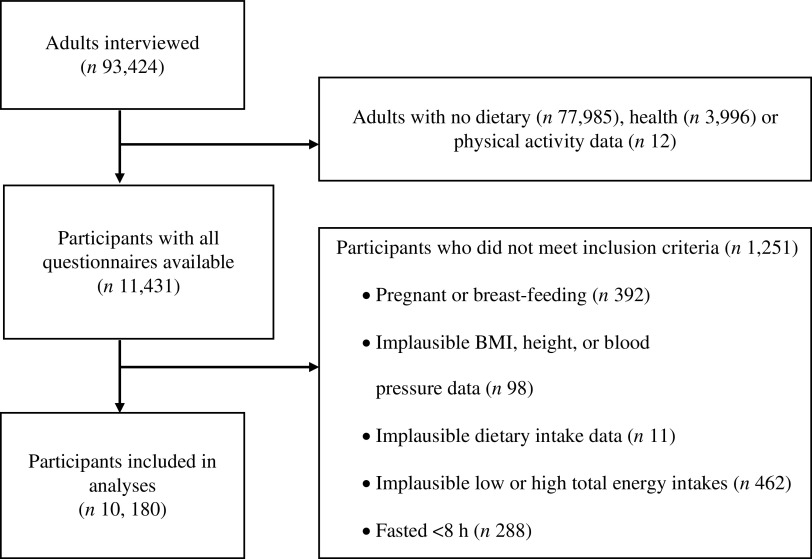
Flow diagram of participants included in a secondary data analysis to examine the association between adherence to the traditional Mexican diet and health outcomes.

Data from 10 180 participants (mean age, 42·8 years; mean BMI, 28·8 kg/m^2^) were analysed (Fig. 1). The mean TMexD index score was 7·0 for the whole sample (range 0–16) and 5·0, 7·5 and 10·0 for the low, medium and high adherence tertiles, respectively. Older individuals, people living in rural areas or in Central and Southern Mexico, those with lower SES or education, and higher physical activity levels and TEI had higher TMexD adherence (Table 1).

**Table 1.** Sociodemographic and health characteristics of 10 180 Mexican adults by tertiles of the traditional Mexican diet index (Mean values with their standard errors)

			Traditional Mexican diet scores‖ (*n* 10 087)						
	Total sample§ (*n* 10 180)		Low 0–6 (*n* 4199)		Medium 7–8 (*n* 3411)		High 9–18 (*n* 2472)		*P* ¶
	Mean	se	Mean	se	Mean	se	Mean	se	
Age (years)	42·8	0·3	41·7	0·5	43·1	0·4	44·4	0·5	0·001
Sex (%)									
Female	57·8	0·9	60·0	1·3	56·1	1·5	56·5	1·7	0·09
Male	42·2	0·9	40·0	1·3	43·9	1·5	43·5	1·7	
Area (%)									
Urban	77·8	0·6	83·7	0·7	74·2	1·2	69·8	1·6	<0·001
Rural	22·2	0·6	16·3	0·7	25·8	1·2	30·2	1·6	
Region									
North	17·1	0·5	21·8	0·9	13·9	0·8	12·0	1·0	<0·001
Centre	51·7	0·8	50·0	1·3	50·8	1·5	54·8	1·8	
South	31·2	0·7	28·2	1·0	35·3	1·4	33·2	1·6	
Socio-economic status (%)									
1st quartile	19·1	0·7	13·7	0·7	22·3	1·2	26·4	1·7	<0·001
2nd quartile	50·4	0·9	50·2	1·3	52·3	1·6	50·0	1·9	
3rd quartile	21·0	0·7	25·6	1·2	17·1	1·2	15·9	1·4	
4th quartile	9·5	0·4	10·5	1·0	8·4	1·2	7·7	1·5	
Education level (%)									
Primary or less	29·2	0·8	24·2	1·1	31·9	1·4	35·7	1·6	<0·001
Secondary school	29·4	0·8	26·7	1·2	32·6	1·6	30·4	1·5	
High school	22·2	0·8	25·7	1·4	19·9	1·3	18·7	1·6	
Higher education	19·2	0·8	23·4	1·1	15·7	1·4	15·2	1·4	
Obesity outcomes									
BMI (kg/m^2^)	28·8	0·1	28·6	0·1	29·0	0·2	28·9	0·2	0·18
Waist circumference (cm)	95·4	0·3	95·2	0·4	95·9	0·4	95·2	0·5	0·44
Diabetes biomarkers									
Glucose (mg/dl)	104·7	0·9	104·4	1·4	104·2	1·3	106·9	1·8	0·43
Glycated Hb (%)	5·7	0·0	5·6	0·0	5·7	0·0	5·8	0·1	0·08
Insulin (μm/ml)	14·9	0·4	16·2	0·7	14·4	0·5	13·3	0·4	0·003
Blood lipids	107·5	0·7	109·2	1·0	106·2	1·0	105·5	1·5	0·03
LDL-cholesterol (mg/dl)	44·1	0·2	44·8	0·3	43·2	0·3	43·9	0·4	0·001
HDL-cholesterol(mg/dl)	142·7	0·7	144·1	1·0	141·8	1·0	141·7	1·5	0·19
Non-HDL-cholesterol (mg/dl)	186·8	0·7	188·9	1·1	185·0	1·1	185·6	1·6	0·02
Total cholesterol (mg/dl)	206·6	2·8	204·0	4·8	209·7	3·9	210·7	5·7	0·57
TAG (mg/dl)	107·5	0·7	109·2	1·0	106·2	1·0	105·5	1·5	0·03
Blood pressure									
Systolic (mmHg)	123·4	0·4	122·2	0·6	124·2	0·6	124·6	0·7	0·01
Diastolic (mmHg)	76·2	0·2	75·7	0·3	76·8	0·3	76·7	0·5	0·05
Diabetes diagnosis* (%)									
Yes	18·2	0·7	17·2	1·1	18·6	1·3	19·3	1·3	0·45
No	81·8	0·7	82·8	1·1	81·4	1·3	80·7	1·3	
Hypertension diagnosis† (%)									
Yes	50·5	1·0	48·4	1·5	53·5	1·5	50·7	1·9	0·05
No	49·5	1·0	51·6	1·5	46·5	1·5	49·3	1·9	
Previous CVD diagnosis‡ (%)									
Yes	3·1	0·3	2·8	0·4	3·4	0·5	2·9	0·5	0·51
No	96·9	0·3	97·2	0·4	96·6	0·5	97·1	0·5	
Physical activity (MET-minutes)	3780·0	63·3	3541·5	97·0	3796·8	101·5	4178·1	128·2	<0·001
Total energy intake (kcal)	2183·4	18·2	2079·5	27·8	2155·8	27·8	2422·8	37·8	<0·001
Smoking status (%)									
Current	17·3	0·6	19·3	1·1	16·1	1·0	14·5	1·3	0·03
Former	19·9	0·7	18·8	1·0	20·4	1·2	21·7	1·4	
Never	62·8	0·8	61·9	1·3	63·5	1·3	63·8	1·8	

MET, metabolic equivalent of task.

* Previous diabetes medical diagnosis or presence of high fasting plasma glucose (≥126 mg/dl or 6.99 mmol/l) or glycated Hb (≥6.5 %) levels.

† Previous hypertension medical diagnosis or presence of high systolic (>130 mmHg) or diastolic (> 80 mmHg) pressure values.

‡ Heart attack, angina and heart failure.

§ Sample sizes: *n* 10 180 except for BMI (*n* 8737), WC (*n* 8716), HbA1c (*n* 9968), insulin (*n* 10 179), LDL-cholesterol (*n* 7826), systolic and diastolic blood pressure (*n* 9077), physical activity (*n* 10 151), total energy intake (*n* 10 154), and smoking (*n* 10 148).

‖ The score ranges refer to the range in the original traditional Mexican diet index, scores in this population ranged from 0 to 16. The scores were calculated as the sum of points across all dietary components in the traditional Mexican diet index, with a higher score indicating a higher adherence.

¶ Calculated using linear regression for continuous variables and Pearson’s *χ* ^2^ tests for categorical variables.

### Intakes of food groups measured in the traditional Mexican diet index

The intake of all fifteen components of the TMexD index differed significantly across the TMexD score tertiles, mainly in an expected direction (Table 2). The percentage of participants following the recommended intakes for each component of the TMexD index was highest for the *tortillas* (70·3 %), herbs and condiments (70·7 %), beverages (72·3 %), and eggs (65·1 %) groups, while lowest for legumes (14·3 %), vegetables (16·8 %), and nuts and seeds (2·9 %).

**Table 2.** Recommended and current intakes of the food groups of 10 180 Mexican adults, according to the traditional Mexican diet index (Mean values with their standard errors)

Food group recommendation in TMexD index		Dietary intakes (g, ml or portion)									Adhering to food group recommendation				
				Traditional Mexican diet scores*							Total sample (*n* 10 180)	Traditional Mexican diet scores*			
		Total sample (*n* 10 180)		Low 0–6 (*n* 4199)		Medium 7–8 (*n* 3411)		High 9–18 (*n* 2472)		*P* †		Low 0–6 (*n* 4199)	Medium 7–8 (*n* 3411)	High 9–18 (*n* 2472)	*P* ‡
		Mean	se	Mean	se	Mean	se	Mean	se			%	%	%	
Maize tortilla	≥4 portion/d	6·9	0·1	5·4	0·1	7·8	0·2	8·5	0·2	<0·001	70·3	49·8	82·6	92·6	<0·001
Legumes	≥100 g/d	48·0	1·0	30·4	0·9	46·4	1·4	85·2	2·7	<0·001	14·3	2·8	12·8	39·4	<0·001
Vegetables	≥240 g/d	138·7	2·8	94·0	2·5	121·4	3·6	242·9	8·0	<0·001	16·8	4·2	13·6	44·0	<0·001
Fruits	≥160 g/d	215·1	4·4	162·5	5·3	208·4	6·5	319·4	11·3	<0·001	45·4	32·1	46·1	67·9	<0·001
Herbs and condiments	≥1 portion/d	2·1	0·0	1·6	0·0	2·3	0·1	2·7	0·1	<0·001	70·7	56·7	76·6	87·7	<0·001
Nuts and seeds	≥30 g/d	3·4	0·3	2·8	0·5	2·5	0·2	5·3	0·6	<0·001	2·9	1·5	1·7	7·0	<0·001
Avocado	≥66 g/d	23·5	1·1	17·5	1·1	23·9	2·1	33·2	2·3	<0·001	20·4	13·8	21·4	30·2	<0·001
Plain water	≥1440 ml/d	1286·2	19·5	1056·8	24·7	1329·7	33·6	1682·6	41·1	<0·001	39·6	26·0	42·5	62·1	<0·001
Beverages	≤240 ml/d	370·1	11·5	440·7	19·5	324·1	18·1	278·8	19·4	<0·001	72·3	65·0	77·5	80·4	<0·001
Other grains	≥100 g/week	144·4	4·3	110·2	4·8	146·3	7·6	203·0	10·8	<0·001	30·0	19·9	32·9	44·8	<0·001
Tubers	≥120 g/week	57·7	1·9	39·3	2·2	57·6	2·7	90·1	4·7	<0·001	18·5	10·9	18·5	32·6	<0·001
Meats	≤240 g/week	600·6	10·3	616·8	14·0	559·2	13·6	627·0	28·9	0·005	24·2	17·1	27·7	32·7	<0·001
Dairy products	≤90 g/week	114·3	3·0	129·4	4·3	94·1	4·7	103·1	6·4	<0·001	63·9	55·8	71·2	71·1	<0·001
Eggs	≤4 portion/week	4·1	0·1	4·5	0·2	3·7	0·2	3·9	0·2	0·001	71·4	65·1	75·0	78·1	<0·001
Maize-based meals	≤1 portion/week	5·0	0·1	5·7	0·2	4·6	0·2	3·9	0·2	<0·001	33·2	21·3	36·3	51·9	<0·001

* The score ranges refer to the range in the original traditional Mexican diet index, and scores in this population ranged from 0 to 16. The scores were calculated as the sum of points across all dietary components in the traditional Mexican diet index, with a higher score indicating a higher adherence.

† Calculated using linear regression.

‡ Calculated using Pearson’s *χ* ^2^ tests.

### Association between the traditional Mexican diet and health outcomes

Results for the association between the TMexD and continuous outcomes are presented in Table 3. In fully adjusted models, high, compared to low, TMexD adherence was associated with lower insulin (−9·8 %; 95 % CI (−16·0, −3·3)), LDL-cholesterol (−4·3 %; 95 % CI (−6·9, −1·5)), non-HDL-cholesterol (−3·9 %; 95 % CI (−6·1, −1·7)) and total cholesterol (−3·5 %; 95 % CI (−5·2, −1·8)) concentrations. Adults with a higher TMexD adherence also had a tendency towards lower HDL-cholesterol (−2·3 %; 95 % CI (−4·2, −0·3)) and higher systolic blood pressure values (1·5 %; 95 % CI (0·2, 2·7)), but these associations were weak. No associations were found between TMexD adherence and any measures of obesity, or with glucose, HbA1c, TAG concentrations or diastolic blood pressure (Table 3). Adherence to the TMexD was not associated with a diagnosis of diabetes, hypertension or CVD (Table 4).

**Table 3.** Percentage differences in non-communicable disease-related outcomes* in adults in the highest tertile *v*. the lowest tertile of adherence† to the traditional Mexican diet (Differences and 95 % confidence intervals)

	Model 1‡			Model 2§			Model 3‖			Model 4¶		
	% Difference	95 % CI	*P* **	% Difference	95 % CI	*P* **	% difference	95 % CI	*P* **	% Difference	95 % CI	*P* **
Obesity measures												
BMI	1·0	−0·6, 2·7	0·22	0·8	−0·9, 2·5	0·36	0·5	−1·1, 2·2	0·54	NA		NA
*n*	8653			8606			8576					
*R* ^2^ ††	0·1			8·2			8·1					
Waist circumference	0·2	−1·0, 1·4	0·79	−0·4	−1·6, 0·7	0·44	−0·6	−1·7, 0·6	0·33	NA		NA
*n*	8631			8580			8551					
*R* ^2^ ††	0·1			10·9			10·8					
Diabetes outcomes												
Glucose	0·9	−1·9, 3·7	0·54	−1·2	−3·6, 1·2	0·31	−1·4	−3·8, 1·0	0·24	−1·3	−3·8, 1·3	0·31
*n*	10 087			9445			9413			8116		
*R* ^2^ ††	0·0			29·0			29·2			28·0		
Glycated Hb	2·1	0·2, 4·0	0·02	0·3	−1·1, 1·7	0·65	0·1	−1·3, 1·6	0·87	0·2	−1·2, 1·6	0·76
*n*	9879			9253			9221			7952		
*R* ^2^ ††	20·0			42·5			42·8			40·7		
Insulin	−10·9	−16·6, −4·9	0·001	−7·5	−13·7, −0·9	0·02	−7·9	−14·1, −1·2	0·02	−9·8	−16·0, −3·3	0·004
*n*	10 086			9444			9412			8115		
*R* ^2^ ††	0·3			5·1			5·2			15·0		
Blood lipids												
LDL-cholesterol	−3·8	−6·8, −0·8	0·01	−3·6	−6·6, −0·5	0·02	−3·5	−6·4, −0·5	0·02	−4·3	−6·9, −1·5	0·002
*n*	7746			6520			6496			5699		
*R* ^2^ ††	0·3			8·6			8·9			9·7		
HDL-cholesterol	−2·2	−4·0, −0·2	0·02	−1·6	−3·6, 0·3	0·10	−2·1	−4·0, −0·2	0·03	−2·3	−4·2, −0·3	0·02
*n*	10 087			8492			8462			7423		
*R* ^2^ ††	0·5			5·7			5·9			10·1		
Non-HDL-cholesterol	−1·7	−4·0, 0·6	0·15	−3·3	−5·5, −0·9	0·007	−3·2	−5·5, −0·9	0·006	−3·9	−6·1, −1·7	0·001
*n*	10 087			8492			8462			7423		
*R* ^2^ ††	0·1			11·0			10·9			14·7		
Total cholesterol	−1·9	−3·7, 0·0	0·05	−2·9	−4·7, −1·0	0·003	−3·0	−4·8, −1·2	0·001	−3·5	−5·2, −1·8	<0·001
*n*	10 087			8492			8462			7423		
*R* ^2^ ††	0·3			9·7			9·7			11·1		
TAG	4·4	−0·8, 9·8	0·09	−1·6	−6·7, 3·8	0·55	−2·2	−7·3, 3·2	0·42	−3·9	−9·2, 1·7	0·17
*n*	10 087			8492			8462			7423		
*R* ^2^ ††	0·1			12·6			12·5			18·5		
Blood pressure												
Systolic	2·2	0·8, 3·6	0·002	0·9	−0·3, 2·1	0·12	0·7	−0·5, 1·8	0·27	1·5	0·2, 2·7	0·01
*R* ^2^ ††	8985			8290			8260			7155		
*n*	0·4			25·5			25·4			23·2		
Diastolic	1·3	−0·2, 2·9	0·08	1·3	−0·2, 2·8	0·08	1·0	−0·5, 2·5	0·21	1·3	−0·4, 2·9	0·12
*n*	8985			8290			8260			7155		
*R* ^2^ ††	0·2			9·4			9·4			15·1		

NA, non-applicable.

* All analyses were conducted through multiple linear regression.

† High adherence reflects individuals with higher scores in the traditional Mexican diet index.

‡ Model 1: unadjusted model.

§ Model 2: adjusted for age, sex, socio-economic status, education level, region of the country, area of residence, physical activity, smoking. Diabetes, blood lipid and blood pressure outcomes were additionally adjusted for family history of disease and use of medication.

‖ Model 3: model 2 plus total energy intake.

¶ Model 4: model 3 plus overweight/obesity status (≥25 kg/m^2^).

** Significance assessed at *P* < 0·004 using the Bonferroni correction.

†† Percent of variance explained by the model.

**Table 4.** OR for having non-communicable disease-related outcomes* in adults in the highest tertile *v*. the lowest tertile of adherence† to the traditional Mexican diet (Odd ratio and 95 % confidence intervals)

	Model 1¶			Model 2**			Model 3††			Model 4‡‡		
	OR	95 % CI	*P* §§	OR	95 % CI	*P* §§	OR	95 % CI	*P* §§	OR	95 % CI	*P* §§
Presence of diabetes‡	1·15	0·92, 1·44	0·21	0·86	0·63, 1·17	0·33	0·89	0·65, 1·21	0·46	0·87	0·62, 1·22	0·40
*n*	9902			8422			8394			7427		
Presence of hypertension§	1·09	0·92, 1·31	0·32	0·97	0·80, 1·19	0·80	0·97	0·79, 1·18	0·75	0·96	0·79, 1·18	0·71
*n*	9159			8454			7268			7268		
Presence of CVD‖	1·02	0·66, 1·58	0·91	0·96	0·60, 1·55	0·87	0·93	0·58, 1·50	0·76	1	0·58, 1·70	0·99
*n*	10 087			9224			9192			7948		

* All analyses were conducted through multiple logistic regression.

† High adherence reflects individuals with higher scores in the traditional Mexican diet index.

‡ Defined as having high fasting glucose (≥126 mg/dl), high glycated Hb levels (≥6·5 %) or a previous diabetes medical diagnosis; total number of cases: 1700.

§ Defined as having either high blood systolic (>130 mmHg) or diastolic (> 80 mmHg) pressure values, or a previous hypertension medical diagnosis; total number of cases: 4751.

‖ Defined as having a previous medical diagnosis of heart attack, angina or heart failure; total number of cases: 332.

¶ Model 1: unadjusted model.

** Model 2: adjusted for age, sex, socio-economic status, education level, region of the country, area of residence, physical activity and smoking. Diabetes, blood lipid and blood pressure outcomes were additionally adjusted for family history of disease and use of medication.

†† Model 3: model 2 plus total energy intake.

‡‡ Model 4: model 3 plus overweight/obesity status (≥25 kg/m^2^).

§§ Significance assessed at *P* < 0·004 using the Bonferroni correction.

### Sensitivity analyses

When performing separate analyses by sex, men with a higher TMexD adherence had greater differences in insulin (−14·0 %; 95 % CI (−23·1, −3·7)), LDL-cholesterol (−7·3 %; 95 % CI (−11·3, −3·0)), non-HDL-cholesterol (−5·1 %; 95 % CI (−8·6, −1·6)) and total cholesterol (−4·7 %; 95 % CI (−7·5, −2·0)) than in the main analyses. Women with high TMexD adherence had a tendency towards lower non-HDL-cholesterol (−2·8 %; 95 % CI (−5·4, −0·1)) and total cholesterol (−2·4 %; 95 % CI (−4·5, −0·2)) only, but these associations were weak (online Supplementary materials II, Table S3). Except for insulin, slightly stronger associations were observed when excluding individuals with an NCD diagnosis (LDL-cholesterol: −5·3 %; non-HDL-cholesterol: −4·4 %; total cholesterol −3·9 %) or individuals dieting after an NCD diagnosis (LDL-cholesterol: −4·8 %; non-HDL-cholesterol: −4·6 %; total cholesterol −4·1 %) (online Supplementary materials II, Table S4). Similar, albeit slightly weaker, associations were observed between a high TMexD adherence and insulin, LDL-cholesterol, non-HDL-cholesterol and total cholesterol when performing multiple imputation (online Supplementary materials II, Table S5). The association between adherence to the TMexD and diagnosis of diabetes, hypertension, or CVD did not differ in any sensitivity analyses (online Supplementary materials II, Table S6–S8).

## Discussion

This study evaluated the associations of adherence to a TMexD with obesity, diabetes and CVD outcomes or risk biomarkers, which are main outcomes of public health concern in Mexico^(19,22)^. To our knowledge, this is the first study to apply a comprehensive, evidence-based index of adherence to the TMexD^(10,11)^ to a nationally representative survey in order to assess associations with health outcomes in Mexican adults.

### Current food intakes according to the traditional Mexican diet index

According to the TMexD index^(10)^, Mexican adults had overall medium adherence to the TMexD, with the mean score being seven out of 18 points, and no individuals reaching the highest score. Higher adherence was reported for *tortillas*, herbs and condiments, beverages, and eggs. In line with previous research^(23,62,65,66,75,76)^, few participants achieved recommendations for legumes, vegetables, and nuts and seeds. However, intakes of some foods might have been slightly underestimated as not all food items present in the TMexD index were assessed in the FFQ used. For instance, foods like amaranth, various herbs and condiments, tubers, and vegetable oils, present in the original index, were not evaluated in ENSANUT. Lastly, three items assessing traditional Mexican food-related habits, present in the original index^(10)^, were omitted as these are not assessed in ENSANUT. This might have led to an underestimation of TMexD adherence in the current sample.

Intakes of foods recommended to be limited were also noteworthy. Few adults (33 %) met the recommendation of maize-based meals (e.g. *tamales*), which are generally energy-dense^(76,77)^. In contrast, many participants (72 %) met the recommended beverage intake, which contrasts with previous research^(62,66,75,76,78)^. Since the index measures traditional drinks only (i.e. *atole*, coffee and *aguas frescas*) but not industrialised beverages (e.g. soda), the percentage of participants meeting the recommendation of energetic beverages is very likely underestimated. Finally, assessing the dairy products group intake was challenging when using this specific index as reference, as a TMexD, as measured by the current index, recommends limiting the consumption of animal-based foods^(10)^ like cheese, but does not establish a limit for yogurt and milk.

### Associations between the traditional Mexican diet and health outcomes

Participants with high TMexD adherence had better outcomes for some diabetes-related biomarkers only. Compared with those with the lowest adherence, they had approximately 10 % lower insulin levels, a biomarker relevant to glucose homoeostasis^(79)^. However, glucose and HbA1c levels did not differ across TMexD tertiles. In previous prospective studies, a Mexican-style diet led to a 14–15 % reduction in insulin values, but not in glucose levels^(30,31)^. This could indicate that these diets improve insulin sensitivity^(80)^, but that further diet or lifestyle factors might need to be tackled to improve glucose levels. The Mexican diet definition in these previous studies, like the TMexD index used in this study, is described as high in beans, maize tortillas, fruits and vegetables but also high in animal fats and full-fat dairy and does not consider items like nuts and seeds, or herbs and condiments. While another cross-sectional study did find that a traditional-style diet was associated with a 51 % reduced odds of having pre-diabetes, a glucose-dependent outcome, the study^(25)^ evaluated a diet with a high fish and low-fat cereal content (which the TMexD index used in the current study does not measure) and was carried out in Comcáac Indians only (as opposed to a nationally representative sample).

Similar results were observed for some blood lipids. Participants in the highest TMexD adherence tertile had about 4 % lower LDL-cholesterol, non-HDL-cholesterol and total cholesterol levels, but no difference in TAG concentrations. Previous prospective studies (defining a Mexican diet as high in beans, maize tortillas, fruits, vegetables, Mexican dishes, animal fats and full-fat dairy products) have observed no changes in TAG in individuals following a Mexican-style diet^(30,31)^. The high fibre content in the TMexD (via fruits, vegetables and legumes^(77,81)^) could explain these results, as they have been suggested to reduce LDL-cholesterol only^(17,82–84)^. However, further studies would need to confirm these claims, as we did not explore the particular macro- or micronutrients that the TMexD is abundant, or low, in. Alternatively, other factors might be relevant for improving TAG concentrations. For instance, obesity can modify the association between diet and triglyceride concentrations^(30,85)^. While this study did adjust for overweight/obesity, no separate analyses were performed by BMI status, which could provide further insights; however, such analyses were beyond the scope of the current study. Intakes of SSB have also been proposed to increase TAG concentrations^(85)^, which is a food group potentially underestimated in the current TMexD index.

It is noteworthy that, apart from total cholesterol, most associations were evident only after adjusting for both TEI and overweight/obesity status, so these differences might be highly dependent on not only TMexD adherence but also on adequate TEI and normal weight.

The associations between the TMexD and lower insulin, LDL-cholesterol, non-HDL-cholesterol and total cholesterol were greater in men, which could be attributed to the higher physical activity levels usually reported among men in the literature^(74,86)^. When excluding participants with no NCD diagnosis, the cholesterol-related associations became stronger, which might indicate that participants modified their diets to one similar to the TMexD after having an NCD diagnosis. Instead, for insulin, diet might only be an important factor in individuals with a disease already in course, like diabetes^(87)^. All associations weakened after multiple imputation, so individuals who self-perceived as having healthy diets or outcomes were potentially more likely to provide complete data^(88)^.

In this study, no benefits of following the TMexD were observed for any obesity, hypertension or CVD (i.e. heart attack, angina or heart failure) outcomes. Previous cross-sectional studies analysing Mexican-style diets (described simply as high in maize foods or as high in *tortillas*, *tacos*, cakes and cookies, SSB, and legumes) have reported equivocal findings for obesity^(24,26)^. As for other indices evaluated in Mexico, a sustainable diet index was inversely associated with obesity in men^(65)^, while the Mexican Alternate Healthy Eating Index was inversely associated with hyperlipidaemia in women with lower educational attainment^(86)^ and lower BMI and waist circumference in men with lower educational attainment^(74)^. Like the TMexD index, these indices promote high intakes of plant-based foods and low intakes of animal source foods. However, the TMexD index, unlike these earlier indices, does not discriminate by the type of meat (e.g. poultry), fat (e.g. polyunsaturated) or grain (e.g. whole grains) consumed. Future studies could evaluate if considering the type of meat, fat or grain modifies the results observed, especially since a high TMexD adherence was associated, albeit weakly, with lower HDL-cholesterol and higher systolic blood pressure values. Future studies could also conduct analyses in adults with lower educational attainment, which seem to have stronger diet–health associations^(74,86)^, possibly given their higher physical activity level or their higher cereal and legumes intake^(74,86,89)^.

### Strengths and limitations

This research studied the associations between the TMexD and an extensive range of NCD risk factors and outcomes in a large and nationally representative sample of Mexican adults. These outcomes were all measured by trained personnel using standardised procedures and clinically relevant parameters^(40)^. The TMexD index used, while still in need of validation, was developed using a systematic review of the evidence^(11)^ and expert consultation^(10)^ to represent a dietary pattern that is objectively traditionally Mexican, including food groups ignored in previous research and not incorporated in earlier indices, like herbs and condiments and nuts and seeds. Findings are relevant to adults residing in Mexico and contribute to the study of traditional diets and indices to measure adherence to traditional diets.

The results presented need to be interpreted considering the study’s limitations. While FFQ are highly valuable for studying habitual diets in epidemiological studies at relatively low costs^(42,90)^, they do not measure all foods consumed, and they can introduce memory recall and social desirability bias^(91,92)^. The FFQ used, while previously validated^(93)^, has been shown to underestimate maize-based meals, potatoes, meat, and legumes, and overestimate tortillas, fruits and vegetables^(45)^, all relevant for calculating TMexD adherence. Likewise, some items present in the TMexD were not evaluated in the FFQ, and thus an adapted version of the originally developed index^(10)^ was used. This issue could have introduced measurement error. For example, vegetable oil, which is included in the TMexD index but not measured in the ENSANUT FFQ, contributes to 4·9 % of the TEI of Mexicans^(60)^. Other non-measured items, such as amaranth, cacao, or native fruits and vegetables like *zapote* and squash blossoms, could also contribute to current diets, although, to our knowledge, no nationally representative studies explore these intakes. Future studies should ideally examine, preferably in prospective studies, the associations with health outcomes of adherence to the full TMexD, as opposed to the adapted version used in the current study. Future research should also evaluate the validity and reliability of the index^(94)^, as this process has not been carried out. Likewise, since few individuals achieved the highest TMexD score range (i.e. ≥12 points out of 18 points), the highest tertile of adherence was constituted by participants with relatively medium scores (i.e. ≥9 points out of 18 points), which might have attenuated the observed associations^(91)^. Moreover, only some assumptions for regression analyses were available for survey data, so models were not tested for issues like influential points, which can affect estimations^(56)^. Lastly, given the cross-sectional nature of this study, it is not possible to claim causality^(95)^ or discard residual confounders or reverse causality bias^(91)^.

Some limitations regarding the index used were also observed. Given that industrialised products (i.e. SSB, salty snacks, desserts, sugars, and cereals with added fats and sugar) considerably contribute to contemporary Mexican diets^(66,75,76,78)^, these might need to be incorporated into the TMexD index as foods whose consumption needs to be limited in order to adhere to a TMexD. The study where the TMexD index was developed theorised that high intakes of healthy plant-based foods would displace non-healthy energy-dense foods (like industrialised products)^(10)^. Nevertheless, this theory could not be tested in the present study, and it might not apply in our sample, as people with higher scores also had higher TEI. Likewise, although current Mexican food guidelines recommend to limit the intake of alcoholic beverages^(77)^, the latter are not measured in the TMexD index, which is similar to other traditional diet indices (e.g. the Nordic or Japanese diet)^(96,97)^. These aspects hinder the ability to classify the TMexD index used as one representing a healthy diet and should be considered in future research. Future studies could explore the relevance of including industrialised products and alcoholic beverages in the TMexD index, as items to be limited, or adjust for their intake in statistical analyses.

Another limitation is that the index contains thresholds for some food group quantities (i.e. herbs and condiments, plain water, nuts and seeds, grains, tubers, dairy products) that did not reach a high consensus amongst the participants who contributed to its development. As such, it is unclear if these thresholds might need some revisions. Similarly, the food groups suggested do not have both lower and upper thresholds of recommended intakes. For instance, the index recommends consuming at least four tortillas per d. However, participants in the highest TMexD tertile consumed an average of eight maize tortillas per d. While maize tortillas are considered a healthy and staple food in Mexico^(77)^, their consumption in exceedingly high amounts might not be optimal, especially since these are not the only grain usually consumed in Mexico^(66)^. In addition, while the geographical region was included as a confounder and although the TMexD index specifically aimed to include foods characteristic of all geographical regions of Mexico^(10,11)^, the differences in food availability and culture across areas could have influenced the level of adherence reached across regions. Future studies should also aim for consistency regarding the geographical area classification in Mexico. For example, previous studies considered central states and Mexico City as separate geographical areas^(76,98)^, whereas others treat them as the same area^(60–62)^. Since the studies used to inform the development of the TMexD index used the North/Centre-Mexico City/South grouping classification (considering Mexico City as part of the central area), we used this latter grouping in the current work. Finally, while multiple imputation was performed, results should be interpreted with caution, particularly for variables where a high percentage of participants had missing data, such as LDL-cholesterol.

### Conclusion

This study evaluated the association of the TMexD with NCD outcomes, which is essential before embarking on promoting this traditional diet or developing interventions to endorse it. Only a small proportion of Mexican adults achieved high TMexD adherence scores, and few met the recommendations for legumes, vegetables, and nuts and seeds. High, compared to low, TMexD adherence was associated with lower concentrations of insulin and some blood lipids (LDL-cholesterol, non-HDL-cholesterol and total cholesterol), but not obesity, diabetes, hypertension or other CVD-related outcomes. Adequate TEI and normal BMI might be required to observe these results, as associations were mostly only evident in models adjusting for these factors. The associations were similar to previous studies evaluating Mexican-style diets, particularly those diets described as high in beans, maize tortillas, fruits and vegetables, even if these were also considered high in animal fats and full-fat dairy products. However, the observed associations in the current work differed from studies describing a Mexican-style diet as high in fish and low-fat cereals. Results must be interpreted with caution due to the study’s limitations, primarily due to the incompatibilities between the TMexD index and the FFQ used, the limited ability of the index to measure industrialised products, and the cross-sectional nature of the study. Moreover, the TMexD index could be modified to improve its compatibility with current health concerns. Specific recommendations to improve the index include dissecting food groups according to public health recommendations (e.g. meat, fat or grain type), adding industrialised products and incorporating an upper limit for tortilla intake.

## Supplementary material

For supplementary material accompanying this paper visit https://doi.org/10.1017/S0007114522002331. click here to view supplementary material
