# Supplementary material for: Adherence to a traditional Mexican diet and non-communicable disease-related outcomes: secondary data analysis of the cross-sectional Mexican National Health and Nutrition Survey
Source: Br J Nutr. 2022 Jul 25;129(7):1266–79. doi: 10.1017/S0007114522002331 (PMC10011591; doi:10.1017/S0007114522002331)
Supplement: Supplementary file 1 [file S0007114522002331sup001.pdf]

Table S1. Classification of food items into the traditional Mexican diet index

Item (points awarded if criteria are met)	Criteria to meet recommendation	Items included in present study
**Food groups**		
Maize products (2)	≥4 *tortillas* or *tostadas*[a,b], or 60 g of *totopos*[a,b] per day	Home-made *nixtamal*[c] or flour *tortilla*, store-bough dough *tortilla*.
Legumes (**2**)	≥1 cup of cooked legumes per day (100 g)	Home-made boiled and refried beans, canned boiled and refried beans, other legumes.
Vegetables (2)	≥3 cups of raw or 1 ½ cup of cooked vegetables per day (240 g)	Tomato, green leaves, *chayote*, carrot, zucchini, broccoli or cauliflower, cabbage, green beans, lettuce, cactus, cucumber, *Poblano* chile, onion, canned vegetables, frozen vegetables, vegetable soup.
Fruits (1)	≥2 cups of fruit per day (160 g)	Banana, *jícama*, orange or tangerine, apple or pear, melon or watermelon, guava, mango, papaya, pineapple, grapefruit, strawberry, grapes, peach.
Beverages (1)	≤1 cup of cacao drinks[b], *atole*[d], or coffee with milk (240 ml), or 2 cups of *aguas frescas*[e] (480 ml) per day	*Atole*[d] with water or milk, coffee with milk, *aguas frescas*[e] with or without sugar.
Herbs and condiments (1)	Cooking[b] with herbs and condiments at least once a day[f]	Consuming onion in sauces or broths, fresh, canned, or dried *chile*, green tomato.
Nuts and seeds (1)	≥30 g per day[f]	Peanuts, fava beans, or pumpkin seeds.
Vegetable fats and oils (1)	≥2/3 avocado (66 g) or 2 teaspoons of vegetable oil[b] per day	Avocado.
Plain water (1)	≥6 glasses per day[f] (1440 ml)	Plain water.
Other grains (1)	≥2 cups of rice (200 g) or toasted amaranth[b] per week[f]	Rice.
Tubers (1)	≥1 ½ large potatoes or cooked tubers per week[f] (120 g)	Boiled and fried potato.
Meats (1)	≤240 g of cooked meats per week	Pork, beef, dried meat, *longaniza* or *chorizo*, ham, or sausage (included when reported in torta, sandwich, and hot dog[g]), chicken thigh or breast, chicken wing or feet, chicken liver or gizzard.
Dairy products (1)	≤90 g of cheese and *requesón*[h] per week[f]	Fresh and full-fat cheese.
Eggs (1)	≤4 eggs per week[f]	Soft or boiled egg, fried egg.
Maize-based meals (1)	≤1 *tamal*[i] or *huarache*[i], 1 *pozole*[j] bowl, 1 *esquite*[i] cup, 1 *elote*[i], 2 *tacos*[i], *quesadillas*[i], *sopes*[i], *gorditas*[i], *tlacoyos*[i], *chalupas*[i] per week	Non-fried and fried no-meat Mexican snacks[k], non-fried and fried Mexican snacks with meat[l], *pozole*[j], *tamal*[i].
**Food-related habits**		
Consuming home-made meals (1)	Consuming meals cooked at home ≥1 per day	Not included (not evaluated in food frequency questionnaire)
Socialising at meals (1)	Eating with family, friends, and colleagues ≥1 per day	Not included (not evaluated in food frequency questionnaire)
Buying foods locally (1)	Buying foods at markets and *tianguis*[m]≥1 per week	Not included (not evaluated in food frequency questionnaire)

[a]Toasted or fried *tortillas* or *tortilla* pieces. [b]Item not measured in food frequency questionnaire used. [c]Traditional process where maize dough has been soaked in an alkaline solution, cooked, and dried. [d]Hot beverage prepared with maize dough. [e]Water blended with fruit/flowers, with or without sugar. [f]This recommended quantity did not reach the consensus in the study conducted to develop the index, it was selected based on plurality of votes (i.e., agreement by a large portion of the sample but less than 50%). [g]Using the standard portion of processed meats (30 g). [h]Type of curd cheese, like fresh cheese. [i]Type of maize-based Mexican snack. [j]Soup made with maize kernels, meat, chile, and seasonings.[k]Includes maize-based Mexican snacks like *sopes*, *quesadillas*, *tlacoyos*, *gorditas*, *enchiladas*. [l]Includes maize-based Mexican snacks like *tacos*, *quesadillas*, *tlacoyos*, *enchiladas*, *gorditas*. [m]Refers to a traditional open-air market, which occurs on certain days of the week.

Table S2. Foods measured in the original traditional Mexican diet index but omitted in the present study, as these are not measured in the Mexican National Health and Nutrition Survey

**Food groups**	**Foods measured in the traditional Mexican diet index food groups but missing in the Mexican National Health and Nutrition Survey**
Maize products	*Tostadas*
Legumes	-
Vegetables	*Chilacayote*, *guaje*, *huitlacoche*, squash blossoms.
Fruits	Anona, *capulín*, *chicozapote*, *mamey*, *nanche*, *pitahaya*, *pitaya*, plum, prickly pear, pomegranate, *tejocote*, *xonocostle*, *zapote*.
Beverages	Cacao drinks.
Herbs and condiments	*Achiote*, *acuyo* or *hoja santa*, *chipilín*, cinnamon, clove, coriander, *epazote*, garlic, parsley, pepper, *tequesquite*, vanilla.
Nuts and seeds	Chia seeds, *chilacayote* seeds, pecan nuts, sesame seeds, sunflower seeds.
Vegetable fats and oils	Vegetable oil.
Other grains	Amaranth.
Plain water	-
Tubers	*Chinchayote*, sweet potato, yucca.
Meats	Chevon, *guajolote* (native turkey), hen, rabbit, turkey.
Dairy products	*Requesón*.
Eggs	*Guajolote* eggs.
Maize-based dishes	*Huaraches*, *chalupas*, *tlayudas*, *esquite*.

Table S3. Percentage differences in non-communicable disease-related outcomes[a] in adults in the highest tertile versus the lowest tertile of adherence[b] to the traditional Mexican diet, women compared to men.

	**Women**								**Men**							
	Model 1*		Model 2†		Model 3‡		Model 4§		Model 1*		Model 2†		Model 3‡		Model 4§	
	% difference (95% CI)	*P* value‖	% difference (95% CI)	*P* value‖	% difference (95% CI)	*P* value‖	% difference (95% CI)	*P* value‖	% difference (95% CI)	*P* value‖	% difference (95% CI)	*P* value‖	% difference (95% CI)	*P* value‖	% difference (95% CI)	*P* value‖
**Obesity measures**																
Body mass index	1.8	0.16	1.2	0.31	1.0	0.41	NA	NA	0.3	0.77	0.6	0.62	0.3	0.80	NA	NA
	(-0.7, 4.3)		(-1.1, 3.6)		(-1.4, 3.5)				(-1.9, 2.6)		(-1.7, 2.8)		(-1.9, 2.5)			
n	4,716		4,688		4,670				3,599		3,580		3,568			
R^2¶	0.2%		9.3%		9.1%				0.1%		8.3%		8.5%			
Waist circumference	0.1	0.91	-0.6	0.43	-0.7	0.39	NA	NA	0.1	0.90	0.0	0.97	-0.2	0.79	NA	NA
	(-1.6, 1.8)		(-2.1, 0.9)		(-2.3, 0.9)				(-1.6, 1.8)		(-1.7, 1.6)		(-1.8, 1.4)			
n	4,701		4,672		4,654				3,592		3,570		3,559			
$R^{2,}$¶	0.0%		10.5%		10.3%				0.1%		13.5%		13.7%			
**Diabetes outcomes**																
Glucose	-0.7	0.72	-0.8	0.61	-0.8	0.63	-0.3	0.84	3.0	0.11	-2.0	0.20	-2.1	0.18	-2.2	0.18
	(-4.4,3.1)		(-4.0, 2.4)		(-4.0, 2.5)		(-3.7, 3.1)		(-0.7, 6.9)		(-5.1, 1.1)		(-5.2, 1.0)		(-5.3, 1.1)	
n	5,518		5,183		5,163		4,440		4,231		3,924		3,912		3,338	
R^2¶	0.0%		32.9%		33.2%		32.8%		0.2%		29.7%		29.7%		28.8%	
Glycated	1.0	0.47	0.8	0.45	0.4	0.74	0.2	0.87	3.9	0.003	-0.4	0.67	-0.3	0.75	0.2	0.85
haemoglobin	(-1.7,3.6)		(-1.3, 3.1)		(-1.9, 2.7)		(-1.9, 2.3)		(1.3, 6.6)		(-2.1, 1.4)		(-2.0, 1.5)		(-1.6, 1.9)	
n	5,381		5,058		5,038		4,337		4,160		3,857		3,845		3,277	
R^2¶	0.1%		42.1%		42.7%		39.3%		0.6%		43.5%		43.4%		43.7%	
Insulin	-6.0	0.13	-5.7	0.17	-5.5	0.20	-7.3	0.08	-16.2	0.001	-10.3	0.05	-11.0	0.04	-14.0	0.009
	(-13.3, 2.0)		(-13.5, 2.7)		(-13.3, 3.0)		(-14.9, 0.9)		(-24.5, -6.9)		(-19.9, 0.4)		(-20.5, -0.3)		(-23.1, -3.7)	
n	5,518		5,183		5,163		4,440		4,230		3,923		3,911		3,337	
R^2¶	0.1%		4.1%		4.3%		11.9%		0.8%		5.9%		6.0%		19.0%	
**Blood lipids**																
LDL-C	-2.2	0.31	-1.6	0.41	-1.7	0.36	-2.0	0.26	-6.2	0.002	-6.4	0.006	-5.7	0.01	-7.3	0.001
	(-6.5, 2.2)		(-5.4, 2.3)		(-5.4, 2.1)		(-5.4, 1.6)		(-9.9, -2.4)		(-10.8, -1.8)		(-9.8, -1.4)		(-11.3, -3.0)	
n	4,463		3,779		3,762		3,289		2,946		2,404		2,397		2,073	
R^2¶	0.2%		14.1%		14.0%		12.0%		0.7%		5.3%		5.8%		9.2%	
HDL-C	-1.4	0.27	-0.7	0.57	-0.9	0.48	-0.9	0.48	-2.5	0.06	-2.9	0.04	-3.5	0.01	-3.4	0.02
	(-4.0, 1.1)		(-3.3, 1.9)		(-3.5, 1.7)		(-3.4, 1.6)		(-5.0, 0.2)		(-5.6, -0.1)		(-6.2, -0.7)		(-6.4, -0.4)	
n	5,518		4,680		4,661		4,052		4,231		3,474		3,463		3,033	
R^2¶	0.6%		5.8%		5.8%		9.0%		0.3%		3.8%		4.4%		10.5%	

Non-HDL-C	-0.8	0.65	-2.3	0.13	-2.4	0.10	-2.8	0.04	-3.1	0.04	-4.1	0.02	-3.8	0.03	-5.1	0.005
	(-4.0, 2.6)		(-5.2, 0.7)		(-5.2, 0.5)		(-5.4, -0.1)		(-6.1, -0.1)		(-7.4, -0.6)		(-7.2, -0.4)		(-8.6, -1.6)	
n	5,518		4,680		4,661		4,052		4,231		3,474		3,463		3,033	
R^2¶	0.0%		16.0%		15.6%		16.2%		0.3%		8.1%		8.2%		13.3%	
Total cholesterol	-1.0	0.48	-1.9	0.12	-2.1	0.07	-2.4	0.03	-3.0	0.01	-3.8	0.006	-3.8	0.007	-4.7	0.001
	(-3.6, 1.8)		(-4.3, 0.5)		(-4.4, 0.2)		(-4.5, -0.2)		(-5.4, -0.6)		(-6.5, -1.1)		(-6.4, -1.1)		(-7.5, -2.0)	
n	5,518		4,680		4,661		4,052		4,231		3,474		3,463		3,033	
R^2¶	0.1%		15.7%		15.6%		14.8%		0.5%		6.1%		6.2%		8.4%	
Triglycerides	4.5	0.19	-2.2	0.49	-2.6	0.44	-4.1	0.21	2.8	0.46	-0.4	0.92	-1.1	0.80	-4.0	0.37
	(-2.3, 11.9)		(-8.4, 4.4)		(-8.8, 4.1)		(-10.3, 2.4)		(-4.4, 10.4)		(-8.7, 8.7)		(-9.4, 8.0)		(-12.2, 5.0)	
n	5,518		4,680		4,661		4,052		4,231		3,474		3,463		3,033	
R^2¶	0.2%		13.2%		13.0%		16.2%		0.0%		9.0%		9.2%		17.3%	
**Blood pressure**																
Systolic	1.7	0.07	0.6	0.44	0.5	0.54	1.5	0.06	2.4	0.004	1.4	0.09	1.1	0.17	1.3	0.13
	(-0.2, 3.6)		(-0.9, 2.1)		(-1.0, 2.0)		(-0.1, 3.2)		(0.8, 4.1)		(-0.2, 3.1)		(-0.5, 2.8)		(-0.4, 3.1)	
n	4,909		4,536		4,517		3,889		3,738		3,416		3,405		2,928	
R^2¶	0.2%		27.5%		27.6%		20.5%		0.6%		15.6%		15.2%		13.9%	
Diastolic	1.6	0.12	1.2	0.18	1.1	0.24	1.6	0.13	0.8	0.40	0.7	0.43	0.3	0.70	0.6	0.52
	(-0.4, 3.6)		(-0.6, 3.1)		(-0.8, 3.1)		(-0.5, 3.7)		(-1.0, 2.5)		(-1.1, 2.5)		(-1.4, 2.1)		(-1.3, 2.5)	
n	4,909		4,536		4,517		3,889		3,738		3,416		3,405		2,928	
R^2¶	0.2%		9.6%		9.5%		12.1%		0.2%		9.2%		9.3%		14.6%	

CI, confidence interval; NA, non-applicable; LDL-C, low-density lipoprotein cholesterol; HDL-C, high-density lipoprotein cholesterol.

[a] All analyses were conducted through multiple linear regression.

[b] High adherence reflects individuals with higher scores in the traditional Mexican diet index.

* Model 1: unadjusted model.

† Model 2: adjusted for age, sex, socioeconomic status, education level, region of the country, area of residence, physical activity, smoking. Diabetes, blood lipid, and blood pressure outcomes were additionally adjusted for family history of disease and use of medication.

‡ Model 3: model 2 plus total energy intake.

§ Model 4: model 3 plus overweight/obesity status (≥25 kg/m2).

|| Significance assessed at $P < 0.004$ using the Bonferroni correction.

¶ Percent of variance explained by the model.

Table S4. Percentage difference in non-communicable disease-related outcomes[a] in the highest tertile versus the lowest tertile of adherence[b] to the traditional Mexican diet, in adults without an NCD diagnosis and adults not dieting after an NCD diagnosis.

	**Participants without NCD diagnosis**								**Participants not dieting after NCD diagnosis**							
	Model 1*		Model 2†		Model 3‡		Model 4§		Model 1*		Model 2†		Model 3‡		Model 4§	
	% difference (95% CI)	*P* value‖	% difference (95% CI)	*P* value‖	% difference (95% CI)	*P* value‖	% difference (95% CI)	*P* value‖	% difference (95% CI)	*P* value‖	% difference (95% CI)	*P* value‖	% difference (95% CI)	*P* value‖	% difference (95% CI)	*P* value‖
**Obesity measures**																
Body mass index	1.0		-0.1		0.0				1.5		0.8		0.5			
	(-1.0, 3.0)	0.34	(-2.0, 1.9)	0.93	(-2.0, 2.0)	0.99	NA	NA	(-0.4, 3.5)	0.13	(-1.1, 2.8)	0.42	(-1.4, 2.5)	0.59	NA	NA
n	5,600		5,565		5,545				6,227		6,189		6,171			
R^2¶	0.1%		8.0%		7.8%				0.2%		9.4%		9.3%			
Waist	0.0		-0.9		-0.9				0.3		-0.5		-0.6			
circumference	(-1.4, 1.4)	0.97	(-2.2, 0.4)	0.19	(-2.2, 0.5)	0.22	NA	NA	(-1.1, 1.7)	0.69	(-1.8, 0.9)	0.48	(-2.0, 0.7)	0.35	NA	NA
n	5,590		5,554		5,534				6,210		6,169		6,151			
R^2¶	0.0%		9.9%		9.7%				0.1%		10.7%		10.6%			
**Diabetes outcomes**																
Glucose	-0.3		-1.4		-1.4		-1.9		0.9		-1.2		-1.5		-2.9	
	(-2.4, 1.8)	0.76	(-3.6, 0.8)	0.20	(-3.6, 0.9)	0.23	(-4.3, 0.5)	0.11	(-2.1, 3.9)	0.57	(-3.6, 1.3)	0.33	(-3.9, 0.9)	0.20	(-5.3, -0.3)	0.02
n	6,120		5,728		5,708		5,230		7,180		6,674		6,654		5,805	
R^2¶	0.1%		6.8%		6.7%		7.7%		0.0%		28.5%		28.8%		27.6%	
Glycated	0.9		-0.1		-0.1		-0.2		1.2		-0.3		-0.7		-0.9	
haemoglobin	(-0.4, 2.2)	0.16	(-1.4, 1.2)	0.86	(-1.3, 1.2)	0.92	(-1.5, 1.2)	0.80	(-0.8, 3.2)	0.25	(-1.9, 1.2)	0.66	(-2.2, 0.9)	0.39	(-2.3, 0.6)	0.23
n	5,998		5,612		5,592		5,124		7,034		6,538		6,518		5,685	
R^2¶	0.4%		12.1%		12.1%		12.3%		0.1%		41.7%		42.5%		40.9%	
Insulin	-10.3		-5.9		-4.8		-7.1		-9.4		-5.1		-5.5		-8.9	
	(-17.0, -3.0)	0.006	(-13.1, 2.0)	0.14	(-12.4, 3.5)	0.25	(-14.2, 0.5)	0.06	(-16.1, -2.1)	0.01	(-12.3, 2.6)	0.18	(-12.8, 2.4)	0.16	(-16.2, -0.9)	0.02
n	6,120		5,728		5,708		5,230		7,179		6,673		6,653		5,804	
R^2¶	0.3%		5.1%		5.3%		16.1%		0.2%		5.7%		5.8%		15.7%	
**Blood lipids**																
LDL-C	-5.0		-5.3		-5.5		-5.3		-4.1		-3.9		-3.9		-4.8	
	(-7.9, -2.0)	0.001	(-8.2, -2.3)	0.001	(-8.5, -2.5)	0.001	(-8.2, -2.3)	0.001	(-7.7, -0.4)	0.03	(-7.4, -0.3)	0.03	(-7.3, -0.4)	0.02	(-7.8, -1.7)	0.002
n	4,907		4,180		4,166		3,830		5,551		4,601		4,586		4,053	
R^2¶	0.5%		10.2%		10.3%		11.8%		0.4%		9.8%		9.8%		11.0%	
HDL-C	-3.1		-2.3		-2.6		-2.3		-1.6		-1.1		-1.8		-2.0	
	(-5.4, -0.7)	0.01	(-4.6, 0.1)	0.06	(-5.0, -0.2)	0.03	(-4.7, 0.1)	0.06	(-4.0, 0.7)	0.17	(-3.4, 1.2)	0.34	(-4.1, 0.5)	0.12	(-4.3, 0.3)	0.09

n	6,120		5,229		5,211		4,805		7,180		5,966		5,949		5,278	
R^2¶	0.7%		5.0%		5.1%		10.8%		0.5%		5.3%		5.5%		10.8%	
Non-HDL-C	-2.6		-4.2		-4.4		-4.4		-2.5		-3.7		-3.7		-4.6	
	(-5.1, -0.1)	0.04	(-6.6, -1.7)	0.001	(-6.9, -1.9)	0.001	(-6.9, -1.9)	0.001	(-5.2, 0.4)	0.08	(-6.5, -0.9)	0.01	(-6.5, -0.9)	0.01	(-7.1, -2.2)	<0.001
n	6,120		5,229		5,211		4,805		7,180		5,966		5,949		5,278	
R^2¶	0.2%		10.8%		10.6%		16.1%		0.2%		11.5%		11.2%		16.1%	
Total cholesterol	-2.8		-3.8		-4.0		-3.9		-2.4		-3.1		-3.4		-4.1	
	(-4.8, -0.8)	0.007	(-5.7, -1.8)	<0.001	(-6.0, -2.0)	<0.001	(-5.9, -1.9)	<0.001	(-4.6, -0.0)	0.04	(-5.4, -0.8)	0.008	(-5.6, -1.1)	0.004	(-6.0, -2.1)	<0.001
n	6,120		5,229		5,211		4,805		7,180		5,966		5,949		5,278	
R^2¶	0.4%		10.0%		10.0%		12.2%		0.4%		10.5%		10.5%		12.2%	
Triglycerides	2.8		-3.3		-3.1		-4.3		1.8		-2.7		-2.7		-5.2	
	(-2.4, 8.4)	0.29	(-8.6, 2.3)	0.24	(-8.5, 2.6)	0.28	(-10.1, 1.8)	0.16	(-3.5, 7.4)	0.50	(-8.2, 3.3)	0.37	(-8.4, 3.4)	0.37	(-11.1, 1.0)	0.09
n	6,120		5,229		5,211		4,805		7,180		5,966		5,949		5,278	
R^2¶	0.2%		10.5%		10.4%		19.3%		0.1%		11.9%		11.8%		18.0%	
**Blood pressure**																
Systolic	2.6		1.3		1.2		1.4		2.1		1.0		0.8		1.5	
	(1.0, 4.2)	0.001	(-0.1, 2.8)	0.07	(-0.3, 2.7)	0.10	(-0.1, 2.9)	0.06	(0.5, 3.8)	0.01	(-0.4, 2.5)	0.14	(-0.6, 2.2)	0.27	(0.1, 3.0)	0.04
n	5,423		4,999		4,980		4,584		6,378		5,834		5,815		5,093	
R^2¶	0.9%		20.9%		20.6%		21.2%		0.5%		26.1%		26.1%		23.8%	
Diastolic	1.6		1.0		0.8		0.9		1.3		1.3		0.9		1.2	
	(-0.2, 3.4)	0.08	(-0.8, 2.8)	0.26	(-1.0, 2.6)	0.40	(-0.9, 2.7)	0.35	(-0.4, 3.0)	0.14	(-0.4, 3.0)	0.13	(-0.8, 2.5)	0.31	(-0.5, 3.0)	0.17
n	5,423		4,999		4,980		4,584		6,378		5,834		5,815		5,093	
R^2¶	0.4%		8.7%		8.6%		13.7%		0.2%		12.0%		12.2%		16.9%	

NCD, non-communicable disease; CI, confidence interval; NA, non-applicable; LDL-C, low-density lipoprotein cholesterol; HDL-C, high-density lipoprotein cholesterol.

[a] All analyses were conducted through multiple linear regression.

[b] High adherence reflects individuals with higher scores in the traditional Mexican diet index.

* Model 1: unadjusted model.

† Model 2: adjusted for age, sex, socioeconomic status, education level, region of the country, area of residence, physical activity, smoking. Diabetes, blood lipid, and blood pressure outcomes were additionally adjusted for family history of disease and use of medication.

‡ Model 3: model 2 plus total energy intake.

§ Model 4: model 3 plus overweight/obesity status (≥25 kg/m2).

|| Significance assessed at $P < 0.004$ using the Bonferroni correction.

¶ Percent of variance explained by the model.

Table S5. Percentage differences in non-communicable disease-related outcomes[a] in 10,087 adults in the highest tertile versus the lowest tertile of adherence[b] to the traditional Mexican diet, data analysed with multiple imputation.

	**Model 1***		**Model 2†**		**Model 3‡**		**Model 4§**	
	% difference (95% CI)	*P* value‖	% difference (95% CI)	*P* value‖	% difference (95% CI)	*P* value‖	% difference (95% CI)	*P* value‖
**Obesity measures**								
Body mass index	0.8 (-1.1, 2.7)	0.41	0.4 (-1.4, 2.3)	0.66	0.3 (-1.6, 2.2)	0.76	NA	NA
Waist circumference	0.2 (-1.2, 1.5)	0.82	-0.5 (-1.7, 0.8)	0.46	-0.6 (-1.8, 0.7)	0.36	NA	NA
**Diabetes outcomes**								
Glucose	1.0 (-1.8, 3.8)	0.50	-0.8 (-3.1, 1.6)	0.51	-0.8 (-3.2, 1.6)	0.49	-0.9 (-3.2, 1.5)	0.47
Glycated haemoglobin	2.1 (0.2, 3.9)	0.02	0.5 (-0.9, 1.8)	0.50	0.4 (-1.0, 1.7)	0.59	0.4 (-1.0, 1.7)	0.60
Insulin	-10.8 (-16.5, -4.8)	0.001	-7.9 (-13.8, -1.5)	0.01	-7.8 (-13.8, -1.5)	0.01	-8.7 (-14.1, -2.9)	0.004
**Blood lipids**								
LDL-C	-3.4 (-6.2, -0.5)	0.02	-3.4 (-6.1, -0.5)	0.02	-3.5 (-6.2, -0.7)	0.01	-3.6 (-6.3, -0.8)	0.01
HDL-C	-2.2 (-4.1, -0.3)	0.02	-1.4 (-3.1, 0.5)	0.14	-1.7 (-3.5, 0.1)	0.06	-1.6 (-3.3, 0.2)	0.09
Non-HDL-C	-1.6 (-4.0, 0.8)	0.17	-2.7 (-4.8, -0.5)	0.01	-2.9 (-5.0, -0.7)	0.01	-3.0 (-5.1, -0.8)	0.007
Total cholesterol	-1.8 (-3.7, 0.1)	0.06	-2.4 (-4.1, -0.6)	0.008	-2.6 (-4.3, -0.9)	0.004	-2.7 (-4.4, -0.9)	0.003
Triglycerides	4.7 (-0.6, 10.2)	0.08	-1.0 (-5.9, 4.1)	0.69	-1.5 (-6.4, 3.6)	0.55	-1.9 (-6.7, 3.1)	0.44
**Blood pressure**								
Systolic	2.4 (1.0, 3.8)	0.001	0.8 (-0.4, 2.0)	0.20	0.8 (-0.5, 2.0)	0.21	0.7 (-0.5, 1.9)	0.25
Diastolic	1.5 (0.0, 3.0)	0.05	0.8 (-0.6, 2.3)	0.25	0.8 (-0.7, 2.2)	0.30	0.6 (-0.7, 2.1)	0.36

CI, confidence interval; NA, non-applicable; LDL-C, low-density lipoprotein cholesterol; HDL-C, high-density lipoprotein cholesterol.

[a] All analyses were conducted through multiple linear regression.

[b] High adherence reflects individuals with higher scores in the traditional Mexican diet index.

* Model 1: unadjusted model.

† Model 2: adjusted for age, sex, socioeconomic status, education level, region of the country, area of residence, physical activity, smoking. Diabetes, blood lipid, and blood pressure outcomes were additionally adjusted for family history of disease and use of medication.

‡ Model 3: model 2 plus total energy intake.

§ Model 4: model 3 plus overweight/obesity status (≥25 kg/m2).

‖ Significance assessed at $P < 0.004$ using the Bonferroni correction.

Table S6. Odds ratio for having non-communicable disease-related outcomes[a] in adults in the highest tertile versus the lowest tertile of adherence[b] to the traditional Mexican diet, women compared to men.

	**Women**								**Men**							
	Model 1*		Model 2†		Model 3‡		Model 4§		Model 1*		Model 2†		Model 3‡		Model 4§	
	OR (95% CI)	*P* value‖	OR (95% CI)	*P* value‖	OR (95% CI)	*P* value‖	OR (95% CI)	*P* value‖	OR (95% CI)	*P* value‖	OR (95% CI)	*P* value‖	OR (95% CI)	*P* value‖	OR (95% CI)	*P* value‖
**Presence of diabetes[c]**	0.86 (0.64, 1.15)	0.30	0.75 (0.48, 1.16)	0.19	0.74 (0.48, 1.15)	0.18	0.7 (0.44, 1.11)	0.12	1.82 (1.32, 2.53)	<0.001	1.07 (0.68, 1.68)	0.78	1.15 (0.74, 1.78)	0.53	1.19 (0.74, 1.92)	0.47
n	5,396		4,529		4,513		4,003		4,168		3,556		3,544		3,087	
**Presence of hypertension[d]**	0.99 (0.77, 1.28)	0.95	0.92 (0.70, 1.20)	0.51	0.93 (0.71, 1.21)	0.57	0.94 (0.71, 1.24)	0.64	1.23 (0.95, 1.58)	0.10	1.03 (0.79, 1.35)	0.83	1.02 (0.78, 1.33)	0.87	0.99 (0.74, 1.34)	0.97
n	5,031		4,652		4,632		3,970		3,790		3,464		3,453		2,960	
**Presence of CVD[e]**	1.04 (0.60, 1.80)	0.90	0.88 (0.47, 1.68)	0.70	0.8 (0.44, 1.45)	0.46	1.0 (0.55, 1.83)	0.99	1.01 (0.52, 1.94)	0.98	1.11 (0.53, 2.32)	0.78	1.14 (0.54, 2.41)	0.72	0.94 (0.37, 2.38)	0.89
n	5,518		5,055		5,035		4,339		4,231		3,831		3,271		3,271	

OR, odds ratio; CI, confidence interval; CVD, cardiovascular disease.

[a] All analyses were conducted through multiple logistic regression.

[b] High adherence reflects individuals with higher scores in the traditional Mexican diet index.

[c] Defined as having high fasting glucose (≥126 mg/dL), high glycated haemoglobin levels (≥6.5%), or a previous diabetes medical diagnosis; total number of cases: 1,700.

[d] Defined as having either high blood systolic (≥130 mmHg) or diastolic (≥ 80 mmHg) pressure values, or a previous hypertension medical diagnosis; total number of cases: 4,751.

[e] Defined as having a previous medical diagnosis of heart attack, angina, or heart failure; total number of cases: 332.

* Model 1: unadjusted model.

† Model 2: adjusted for age, sex, socioeconomic status, education level, region of the country, area of residence, physical activity, smoking. Diabetes, blood lipid, and blood pressure outcomes were additionally adjusted for family history of disease and use of medication.

‡ Model 3: model 2 plus total energy intake.

§ Model 4: model 3 plus overweight/obesity status (≥25 kg/m2).

‖ Significance assessed at $P < 0.004$ using the Bonferroni correction.

Table S7. Odds ratio for having non-communicable disease-related outcomes[a] in the highest tertile versus the lowest tertile of adherence[b] to the traditional Mexican diet, in adults without an NCD diagnosis and adults not dieting after an NCD diagnosis.

	**Participants without NCD diagnosis**								**Participants not dieting after NCD diagnosis**							
	Model 1*		Model 2†		Model 3‡		Model 4§		Model 1*		Model 2†		Model 3‡		Model 4§	
	OR (95% CI)	*P* value‖	OR (95% CI)	*P* value‖	OR (95% CI)	*P* value‖	OR (95% CI)	*P* value‖	OR (95% CI)	*P* value‖	OR (95% CI)	*P* value‖	OR (95% CI)	*P* value‖	OR (95% CI)	*P* value‖
**Presence of diabetes[c]**	1.23 (0.82, 1.84)	0.32	1.07 (0.70, 1 .64)	0.75	1.13 (0.74, 1.71)	0.57	1.08 (0.68, 1.72)	0.73	1.08 (0.82, 1.43)	0.58	0.79 (0.54, 1.14)	0.20	0.82 (0.57, 1.17)	0.27	0.78 (0.53, 1.16)	0.22
n	5,998		5,612		5,592		5,124		7,044		6,149		6,131		5,442	
**Presence of hypertension[d]**	1.23 (0.99, 1.53)	0.06	1.09 (0.87, 1 .37)	0.43	1.10 (0.88, 1.38)	0.40	1.08 (0.85, 1.38)	0.51	1.18 (0.95, 1.46)	0.14	1.11 (0.88, 1.40)	0.38	1.08 (0.86, 1.37)	0.50	1.05 (0.84, 1.32)	0.65
n	5,423		4,999		4,980		4,584		6,482		5,934		5,914		5,161	
**Presence of CVD[e]**	NA	NA	NA	NA	NA	NA	NA	NA	0.99 (0.57, 1.72)	0.96	0.8 (0.43, 1.52)	0.50	0.84 (0.46, 1.54)	0.57	0.92 (0.47, 1.81)	0.80
n									7,180		6,515		6,495		5,673	

NCD, non-communicable disease; OR, odds ratio; CI, confidence interval; CVD, cardiovascular disease; NA, non-applicable.

[a] All analyses were conducted through multiple logistic regression.

[b] High adherence reflects individuals with higher scores in the traditional Mexican diet index.

[c] Defined as having high fasting glucose (≥126 mg/dL), high glycated haemoglobin levels (≥6.5%), or a previous diabetes medical diagnosis; total number of cases: 1,700.

[d] Defined as having either high blood systolic (≥130 mmHg) or diastolic (≥ 80 mmHg) pressure values, or a previous hypertension medical diagnosis; total number of cases: 4,751.

[e] Defined as having a previous medical diagnosis of heart attack, angina, or heart failure; total number of cases: 332.

* Model 1: unadjusted model.

† Model 2: adjusted for age, sex, socioeconomic status, education level, region of the country, area of residence, physical activity, smoking. Diabetes, blood lipid, and blood pressure outcomes were additionally adjusted for family history of disease and use of medication.

‡ Model 3: model 2 plus total energy intake.

§ Model 4: model 3 plus overweight/obesity status (≥25 kg/m2).

‖ Significance assessed at $P < 0.004$ using the Bonferroni correction.

Table S8. Odds ratio for having non-communicable disease-related outcomes[a] in 10,087 adults in the highest tertile versus the lowest tertile of adherence[b] to the traditional Mexican diet, data analysed with multiple imputation.

	**Model 1***		**Model 2†**		**Model 3‡**		**Model 4§**	
	OR (95% CI)	*P* value‖	OR (95% CI)	*P* value‖	OR (95% CI)	*P* value‖	OR (95% CI)	*P* value‖
**Presence of diabetes**[c]	1.16 (0.93, 1.45)	0.20	0.96 (0.71, 1.29)	0.76	1.01 (0.75, 1.37)	0.93	1.01 (0.75, 1.37)	0.93
**Presence of hypertension**[d]	1.12 (0.93, 1.34)	0.23	0.96 (0.78, 1.17)	0.67	0.98 (0.80, 1.19)	0.81	0.96 (0.79, 1.17)	0.70
**Presence of CVD**[e]	1.02 (0.65, 1.62)	0.92	1.04 (0.65, 1.67)	0.87	1.08 (0.67, 1.73)	0.76	1.07 (0.66, 1.72)	0.78

OR, odds ratio; CI, confidence interval; CVD, cardiovascular disease.

[a] All analyses were conducted through multiple logistic regression.

[b] High adherence reflects individuals with higher scores in the traditional Mexican diet index.

[c] Defined as having high fasting glucose (≥126 mg/dL), high glycated haemoglobin levels (≥6.5%), or a previous diabetes medical diagnosis; total number of cases: 1,700.

[d] Defined as having either high blood systolic (≥130 mmHg) or diastolic (≥ 80 mmHg) pressure values, or a previous hypertension medical diagnosis; total number of cases: 4,751.

[e] Defined as having a previous medical diagnosis of heart attack, angina, or heart failure; total number of cases: 332.

* Model 1: unadjusted model.

† Model 2: adjusted for age, sex, socioeconomic status, education level, region of the country, area of residence, physical activity, smoking. Diabetes, blood lipid, and blood pressure outcomes were additionally adjusted for family history of disease and use of medication.

‡ Model 3: model 2 plus total energy intake.

§ Model 4: model 3 plus overweight/obesity status (≥25 kg/m2).

‖ Significance assessed at *P* < 0.004 using the Bonferroni correction.
